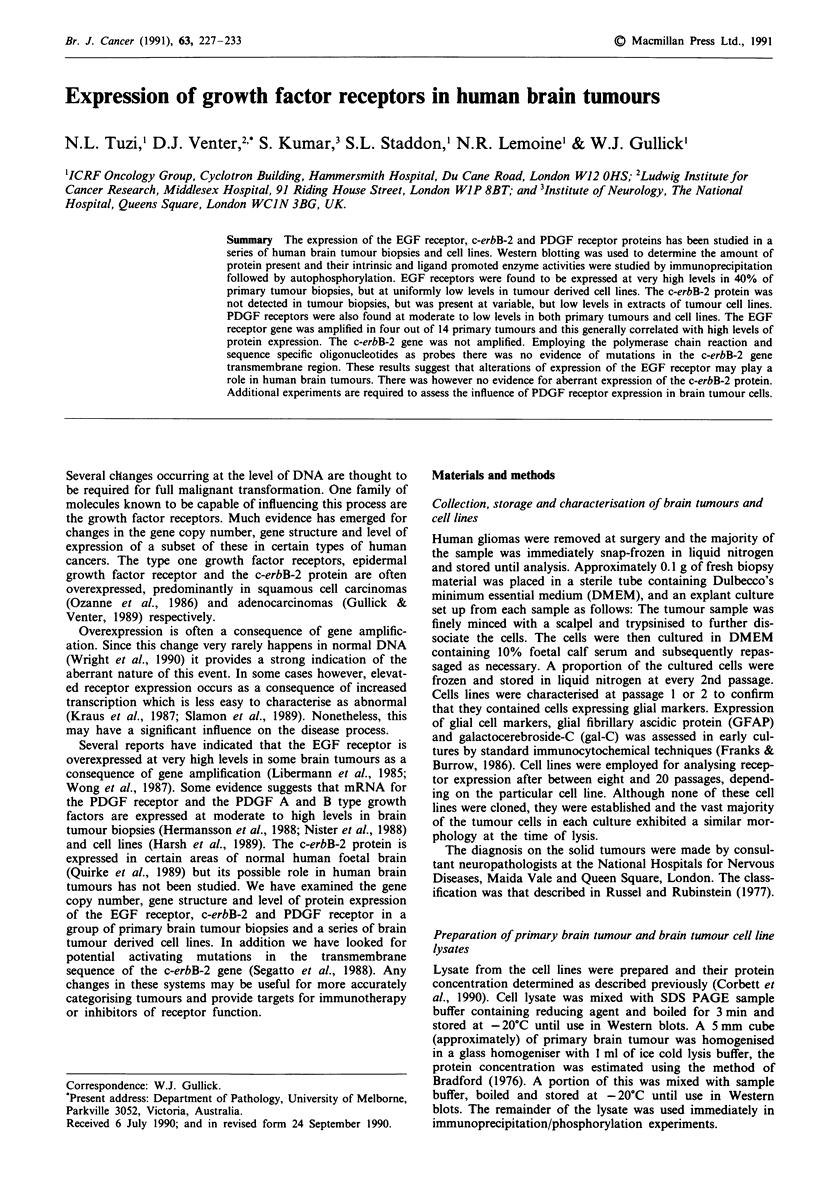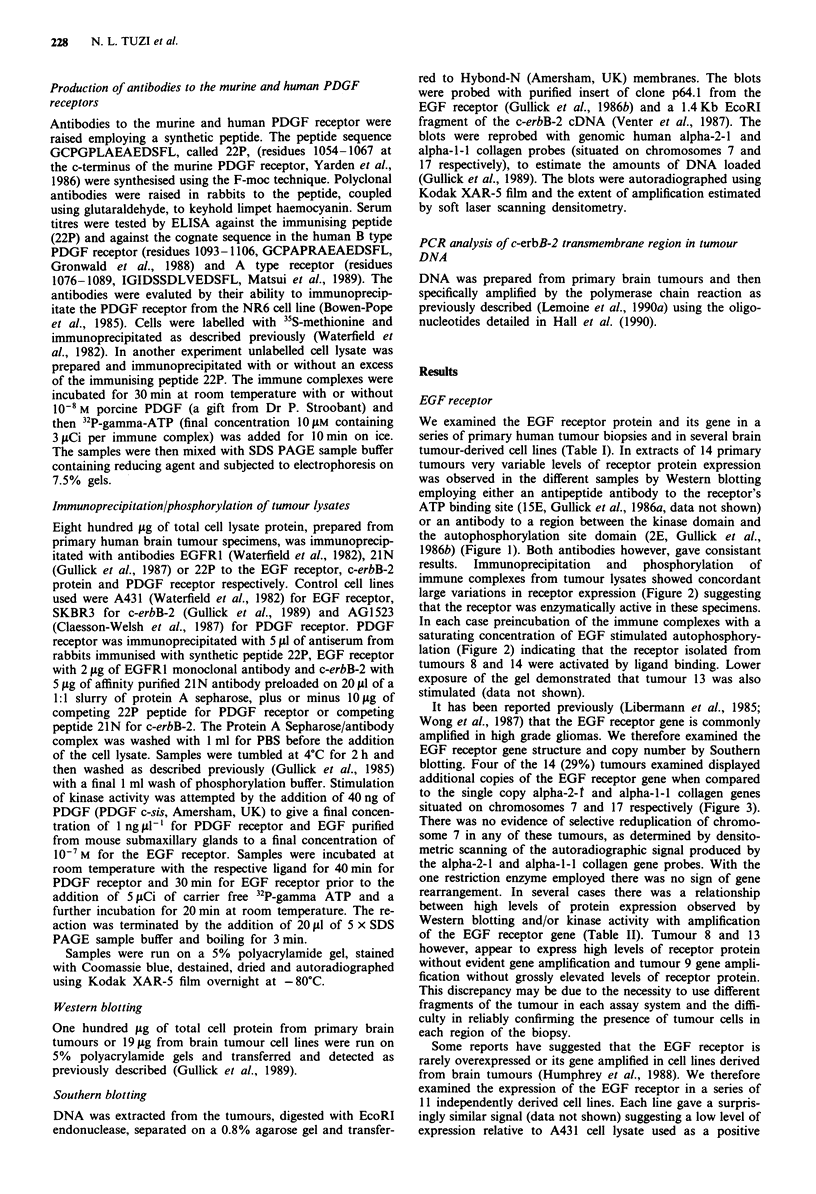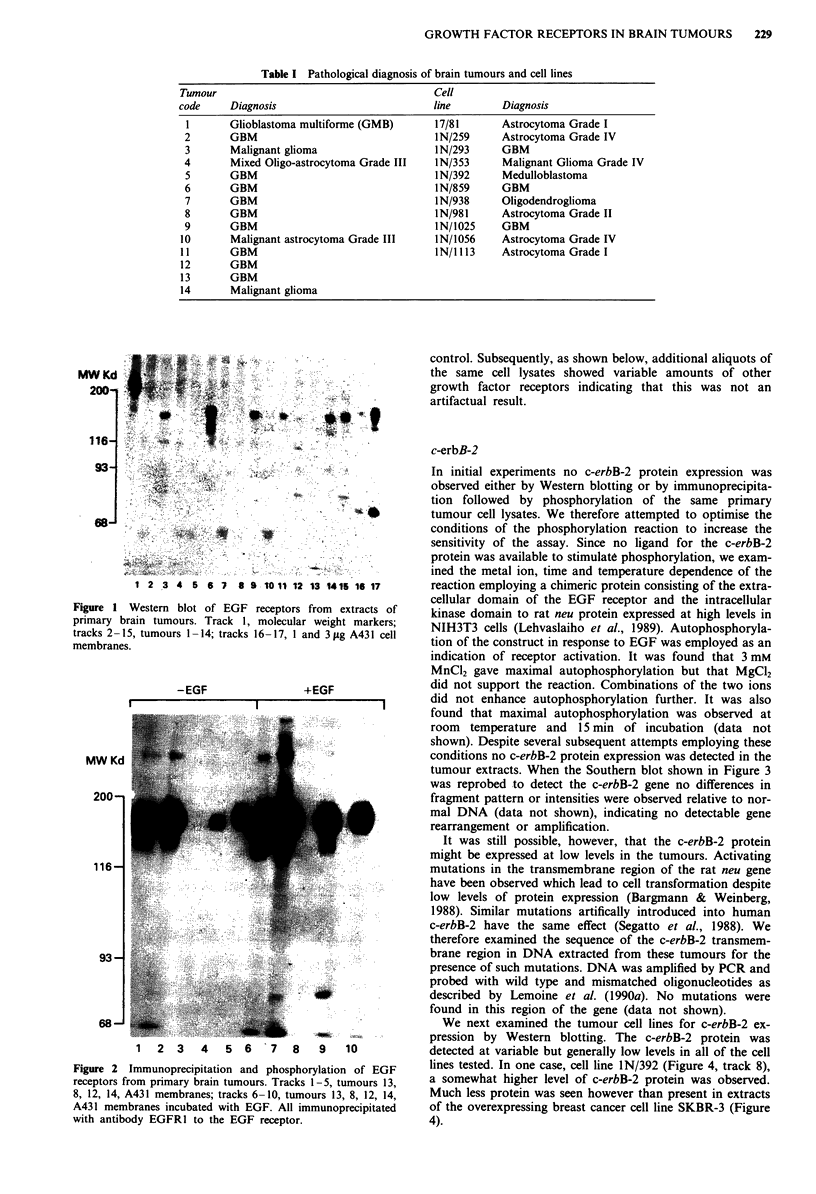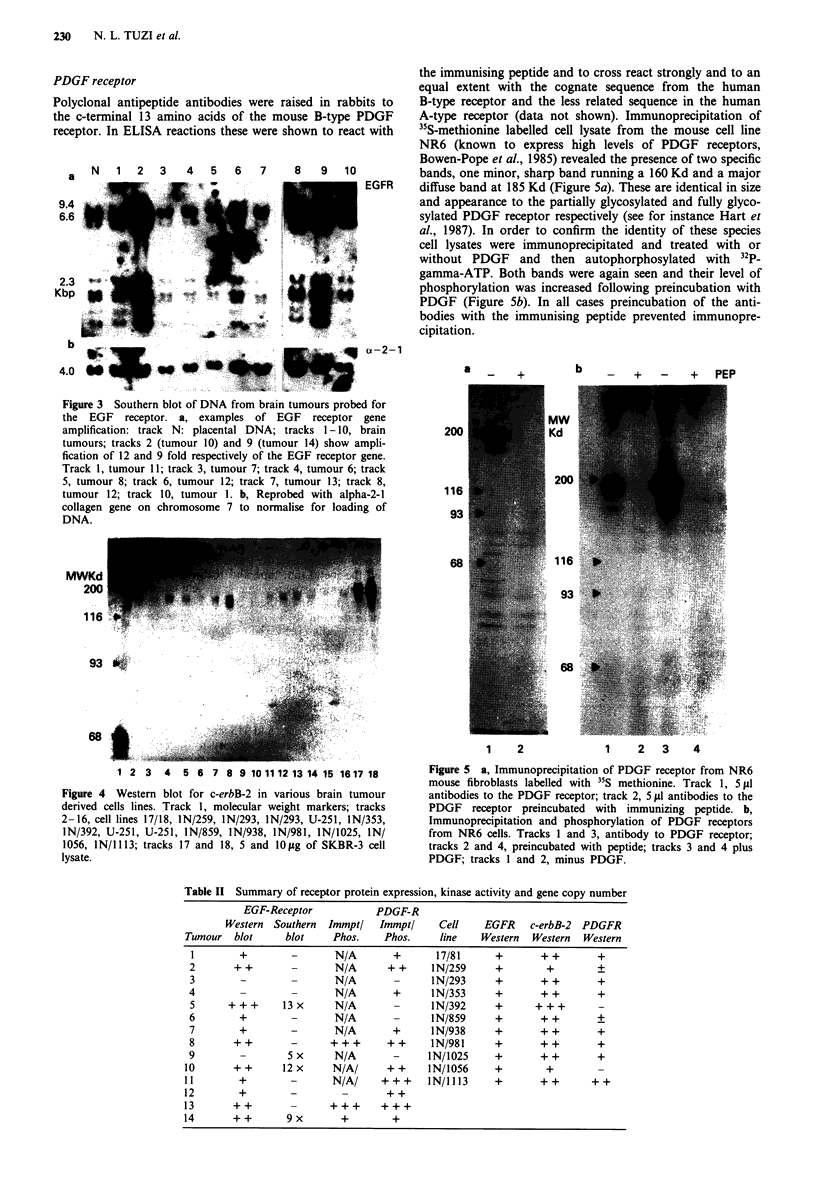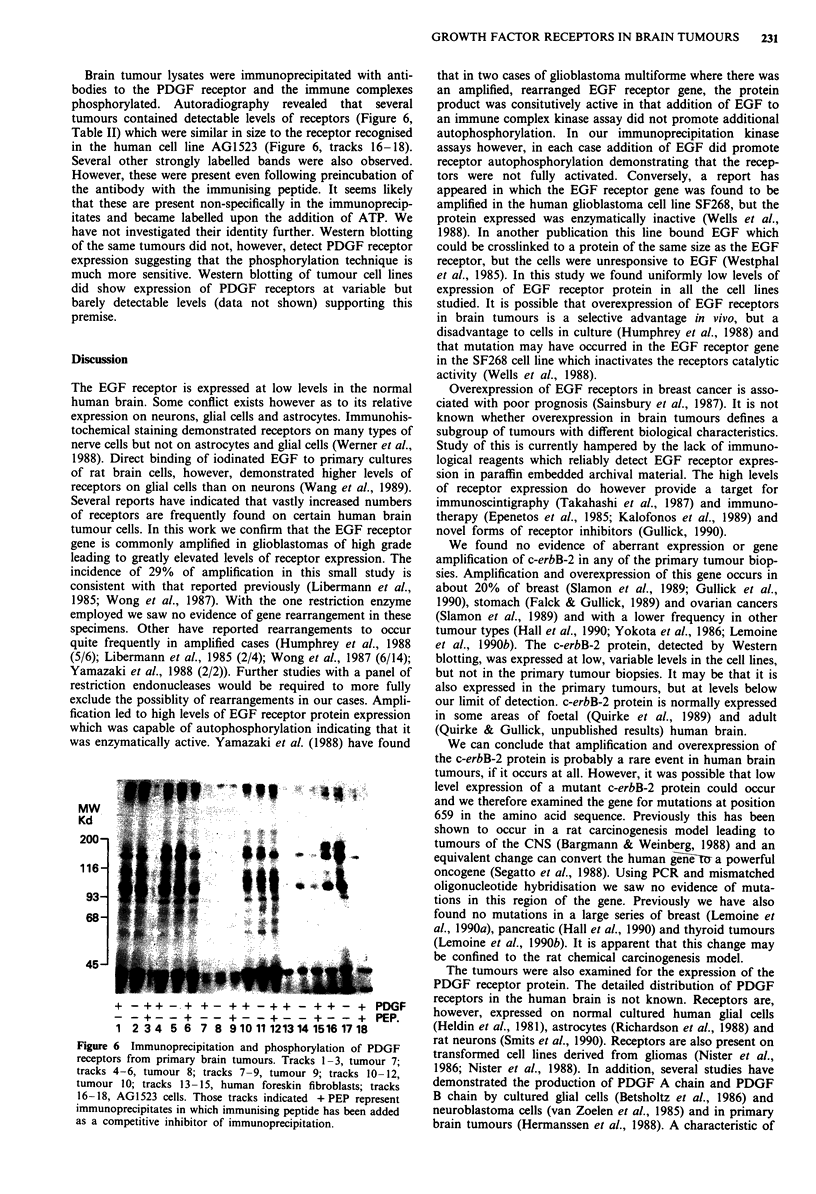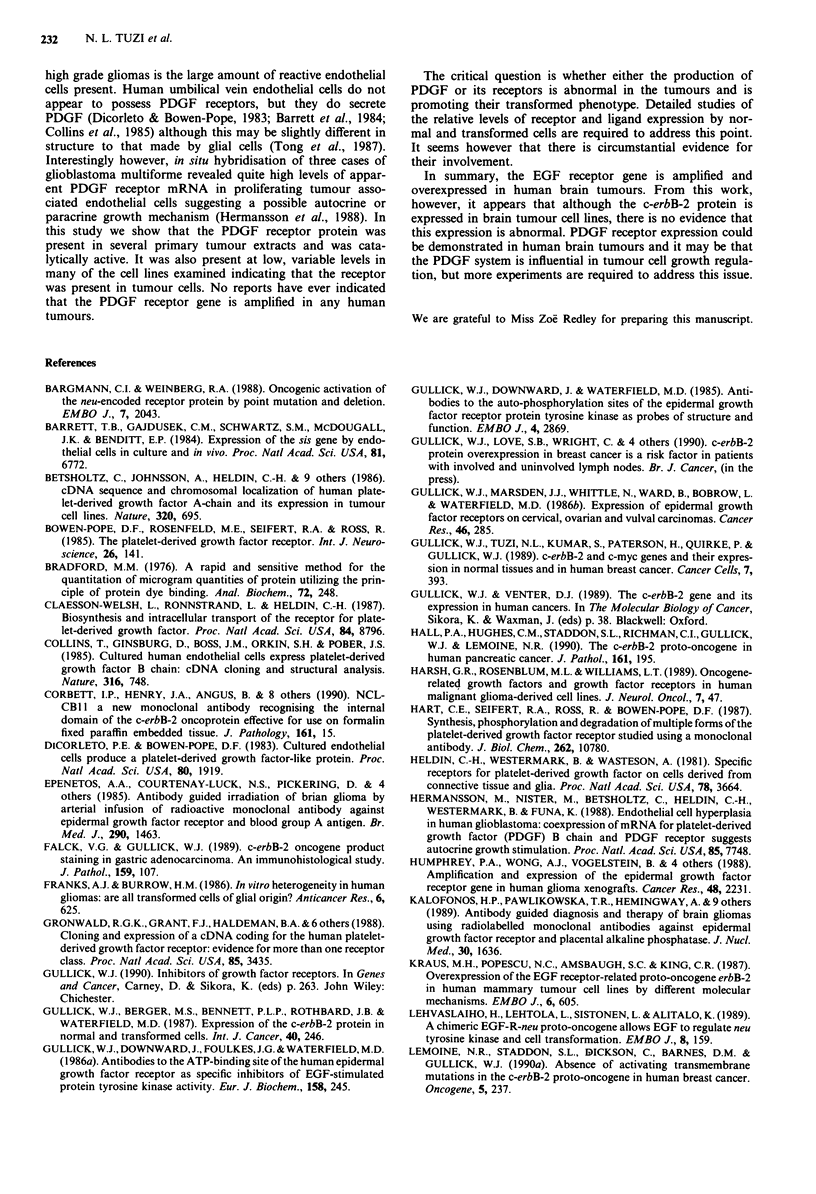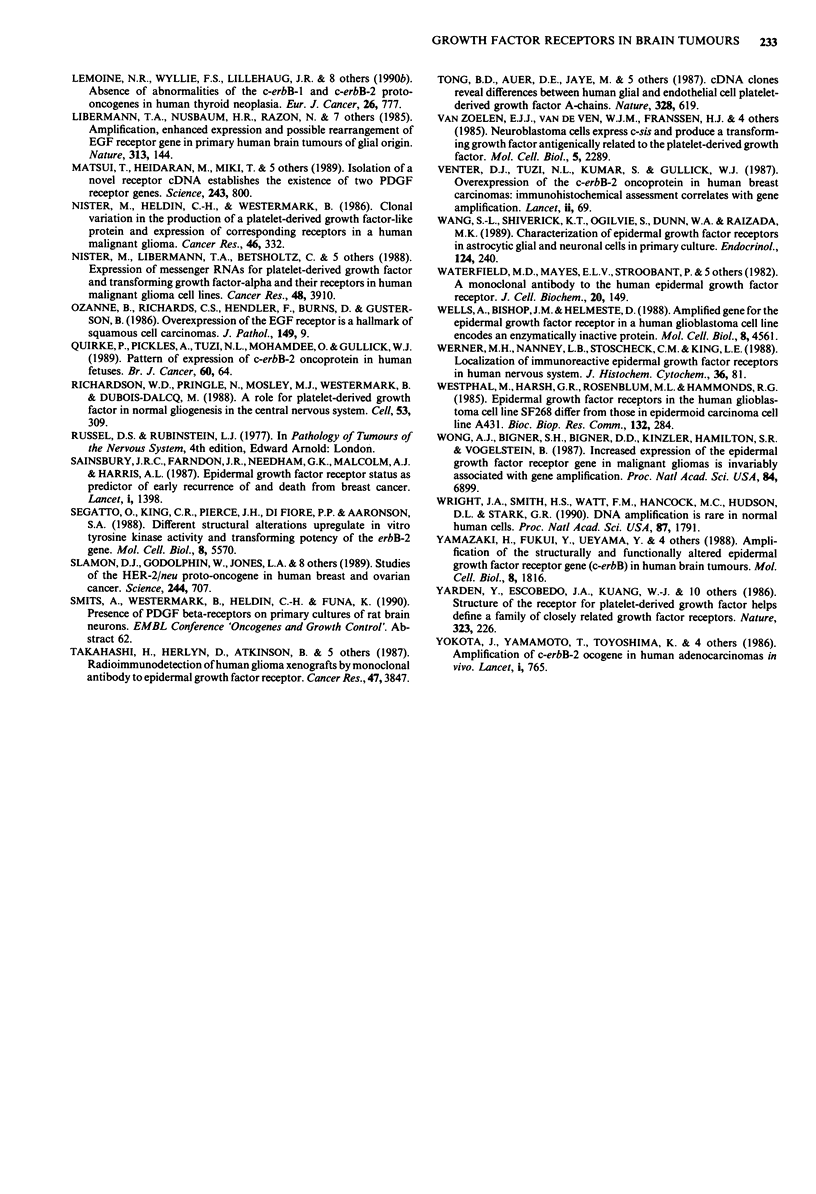# Expression of growth factor receptors in human brain tumours.

**DOI:** 10.1038/bjc.1991.54

**Published:** 1991-02

**Authors:** N. L. Tuzi, D. J. Venter, S. Kumar, S. L. Staddon, N. R. Lemoine, W. J. Gullick

**Affiliations:** ICRF Oncology Group, Hammersmith Hospital, London, UK.

## Abstract

**Images:**


Br. J. Cancer (1991), 63, 227-233 © Macmillan Press Ltd., 1991

### Expression of growth factor receptors in human brain tumours

N.L. Tuzi,[1] D.J. Venter,[2,*] S. Kumar,[3] S.L. Staddon,[1] N.R. Lemoine[1] & W.J. Gullick[1]

[1]*ICRF Oncology Group, Cyclotron Building, Hammersmith Hospital, Du Cane Road, London W12 0HS;* [2]*Ludwig Institute for Cancer Research, Middlesex Hospital, 91 Riding House Street, London W1P 8BT; and* [3]*Institute of Neurology, The National Hospital, Queens Square, London WC1N 3BG, UK.*

**Summary** The expression of the EGF receptor, c-*erb*B-2 and PDGF receptor proteins has been studied in a series of human brain tumour biopsies and cell lines. Western blotting was used to determine the amount of protein present and their intrinsic and ligand promoted enzyme activities were studied by immunoprecipitation followed by autophosphorylation. EGF receptors were found to be expressed at very high levels in 40% of primary tumour biopsies, but at uniformly low levels in tumour derived cell lines. The c-*erb*B-2 protein was not detected in tumour biopsies, but was present at variable, but low levels in extracts of tumour cell lines. PDGF receptors were also found at moderate to low levels in both primary tumours and cell lines. The EGF receptor gene was amplified in four out of 14 primary tumours and this generally correlated with high levels of protein expression. The c-*erb*B-2 gene was not amplified. Employing the polymerase chain reaction and sequence specific oligonucleotides as probes there was no evidence of mutations in the c-*erb*B-2 gene transmembrane region. These results suggest that alterations of expression of the EGF receptor may play a role in human brain tumours. There was however no evidence for aberrant expression of the c-*erb*B-2 protein. Additional experiments are required to assess the influence of PDGF receptor expression in brain tumour cells.

Several changes occurring at the level of DNA are thought to be required for full malignant transformation. One family of molecules known to be capable of influencing this process are the growth factor receptors. Much evidence has emerged for changes in the gene copy number, gene structure and level of expression of a subset of these in certain types of human cancers. The type one growth factor receptors, epidermal growth factor receptor and the c-*erb*B-2 protein are often overexpressed, predominantly in squamous cell carcinomas (Ozanne *et al.*, 1986) and adenocarcinomas (Gullick & Venter, 1989) respectively.

Overexpression is often a consequence of gene amplification. Since this change very rarely happens in normal DNA (Wright *et al.*, 1990) it provides a strong indication of the aberrant nature of this event. In some cases however, elevated receptor expression occurs as a consequence of increased transcription which is less easy to characterise as abnormal (Kraus *et al.*, 1987; Slamon *et al.*, 1989). Nonetheless, this may have a significant influence on the disease process.

Several reports have indicated that the EGF receptor is overexpressed at very high levels in some brain tumours as a consequence of gene amplification (Libermann *et al.*, 1985; Wong *et al.*, 1987). Some evidence suggests that mRNA for the PDGF receptor and the PDGF A and B type growth factors are expressed at moderate to high levels in brain tumour biopsies (Hermansson *et al.*, 1988; Nister *et al.*, 1988) and cell lines (Harsh *et al.*, 1989). The c-*erb*B-2 protein is expressed in certain areas of normal human foetal brain (Quirke *et al.*, 1989) but its possible role in human brain tumours has not been studied. We have examined the gene copy number, gene structure and level of protein expression of the EGF receptor, c-*erb*B-2 and PDGF receptor in a group of primary brain tumour biopsies and a series of brain tumour derived cell lines. In addition we have looked for potential activating mutations in the transmembrane sequence of the c-*erb*B-2 gene (Segatto *et al.*, 1988). Any changes in these systems may be useful for more accurately categorising tumours and provide targets for immunotherapy or inhibitors of receptor function.

Correspondence: W.J. Gullick.
*Present address: Department of Pathology, University of Melborne, Parkville 3052, Victoria, Australia.
Received 6 July 1990; and in revised form 24 September 1990.

### Materials and methods

### *Collection, storage and characterisation of brain tumours and cell lines*

Human gliomas were removed at surgery and the majority of the sample was immediately snap-frozen in liquid nitrogen and stored until analysis. Approximately 0.1 g of fresh biopsy material was placed in a sterile tube containing Dulbecco's minimum essential medium (DMEM), and an explant culture set up from each sample as follows: The tumour sample was finely minced with a scalpel and trypsinised to further dissociate the cells. The cells were then cultured in DMEM containing 10% foetal calf serum and subsequently repassaged as necessary. A proportion of the cultured cells were frozen and stored in liquid nitrogen at every 2nd passage. Cells lines were characterised at passage 1 or 2 to confirm that they contained cells expressing glial markers. Expression of glial cell markers, glial fibrillary ascidic protein (GFAP) and galactocerebroside-C (gal-C) was assessed in early cultures by standard immunocytochemical techniques (Franks & Burrow, 1986). Cell lines were employed for analysing receptor expression after between eight and 20 passages, depending on the particular cell line. Although none of these cell lines were cloned, they were established and the vast majority of the tumour cells in each culture exhibited a similar morphology at the time of lysis.

The diagnosis on the solid tumours were made by consultant neuropathologists at the National Hospitals for Nervous Diseases, Maida Vale and Queen Square, London. The classification was that described in Russel and Rubinstein (1977).

### *Preparation of primary brain tumour and brain tumour cell line lysates*

Lysate from the cell lines were prepared and their protein concentration determined as described previously (Corbett *et al.*, 1990). Cell lysate was mixed with SDS PAGE sample buffer containing reducing agent and boiled for 3 min and stored at −20°C until use in Western blots. A 5 mm cube (approximately) of primary brain tumour was homogenised in a glass homogeniser with 1 ml of ice cold lysis buffer, the protein concentration was estimated using the method of Bradford (1976). A portion of this was mixed with sample buffer, boiled and stored at −20°C until use in Western blots. The remainder of the lysate was used immediately in immunoprecipitation/phosphorylation experiments.

*Production of antibodies to the murine and human PDGF receptors*

Antibodies to the murine and human PDGF receptor were raised employing a synthetic peptide. The peptide sequence GCPGPLAEAEDSFL, called 22P, (residues 1054-1067 at the c-terminus of the murine PDGF receptor, Yarden *et al.*, 1986) were synthesised using the F-moc technique. Polyclonal antibodies were raised in rabbits to the peptide, coupled using glutaraldehyde, to keyhold limpet haemocyanin. Serum titres were tested by ELISA against the immunising peptide (22P) and against the cognate sequence in the human B type PDGF receptor (residues 1093-1106, GCPAPRAEAEDSFL, Gronwald *et al.*, 1988) and A type receptor (residues 1076-1089, IGIDSSDLVEDSFL, Matsui *et al.*, 1989). The antibodies were evaluted by their ability to immunoprecipitate the PDGF receptor from the NR6 cell line (Bowen-Pope *et al.*, 1985). Cells were labelled with $^{35}$S-methionine and immunoprecipitated as described previously (Waterfield *et al.*, 1982). In another experiment unlabelled cell lysate was prepared and immunoprecipitated with or without an excess of the immunising peptide 22P. The immune complexes were incubated for 30 min at room temperature with or without $10^{-8}$ M porcine PDGF (a gift from Dr P. Stroobant) and then $^{32}$P-gamma-ATP (final concentration 10 $\mu$M containing 3 $\mu$Ci per immune complex) was added for 10 min on ice. The samples were then mixed with SDS PAGE sample buffer containing reducing agent and subjected to electrophoresis on 7.5% gels.

*Immunoprecipitation/phosphorylation of tumour lysates*

Eight hundred $\mu$g of total cell lysate protein, prepared from primary human brain tumour specimens, was immunoprecipitated with antibodies EGFR1 (Waterfield *et al.*, 1982), 21N (Gullick *et al.*, 1987) or 22P to the EGF receptor, c-*erb*B-2 protein and PDGF receptor respectively. Control cell lines used were A431 (Waterfield *et al.*, 1982) for EGF receptor, SKBR3 for c-*erb*B-2 (Gullick *et al.*, 1989) and AG1523 (Claesson-Welsh *et al.*, 1987) for PDGF receptor. PDGF receptor was immunoprecipitated with 5 $\mu$l of antiserum from rabbits immunised with synthetic peptide 22P, EGF receptor with 2 $\mu$g of EGFR1 monoclonal antibody and c-*erb*B-2 with 5 $\mu$g of affinity purified 21N antibody preloaded on 20 $\mu$l of a 1:1 slurry of protein A sepharose, plus or minus 10 $\mu$g of competing 22P peptide for PDGF receptor or competing peptide 21N for c-*erb*B-2. The Protein A Sepharose/antibody complex was washed with 1 ml for PBS before the addition of the cell lysate. Samples were tumbled at 4°C for 2 h and then washed as described previously (Gullick *et al.*, 1985) with a final 1 ml wash of phosphorylation buffer. Stimulation of kinase activity was attempted by the addition of 40 ng of PDGF (PDGF c-*sis*, Amersham, UK) to give a final concentration of 1 ng $\mu$l$^{-1}$ for PDGF receptor and EGF purified from mouse submaxillary glands to a final concentration of $10^{-7}$ M for the EGF receptor. Samples were incubated at room temperature with the respective ligand for 40 min for PDGF receptor and 30 min for EGF receptor prior to the addition of 5 $\mu$Ci of carrier free $^{32}$P-gamma ATP and a further incubation for 20 min at room temperature. The reaction was terminated by the addition of 20 $\mu$l of 5 × SDS PAGE sample buffer and boiling for 3 min.

Samples were run on a 5% polyacrylamide gel, stained with Coomassie blue, destained, dried and autoradiographed using Kodak XAR-5 film overnight at −80°C.

*Western blotting*

One hundred $\mu$g of total cell protein from primary brain tumours or 19 $\mu$g from brain tumour cell lines were run on 5% polyacrylamide gels and transferred and detected as previously described (Gullick *et al.*, 1989).

*Southern blotting*

DNA was extracted from the tumours, digested with EcoRI endonuclease, separated on a 0.8% agarose gel and transferred to Hybond-N (Amersham, UK) membranes. The blots were probed with purified insert of clone p64.1 from the EGF receptor (Gullick *et al.*, 1986*b*) and a 1.4 Kb EcoRI fragment of the c-*erb*B-2 cDNA (Venter *et al.*, 1987). The blots were reprobed with genomic human alpha-2-1 and alpha-1-1 collagen probes (situated on chromosomes 7 and 17 respectively), to estimate the amounts of DNA loaded (Gullick *et al.*, 1989). The blots were autoradiographed using Kodak XAR-5 film and the extent of amplification estimated by soft laser scanning densitometry.

*PCR analysis of c-*erb*B-2 transmembrane region in tumour DNA*

DNA was prepared from primary brain tumours and then specifically amplified by the polymerase chain reaction as previously described (Lemoine *et al.*, 1990*a*) using the oligonucleotides detailed in Hall *et al.* (1990).

### Results

*EGF receptor*

We examined the EGF receptor protein and its gene in a series of primary human tumour biopsies and in several brain tumour-derived cell lines (Table I). In extracts of 14 primary tumours very variable levels of receptor protein expression was observed in the different samples by Western blotting employing either an antipeptide antibody to the receptor's ATP binding site (15E, Gullick *et al.*, 1986*a*, data not shown) or an antibody to a region between the kinase domain and the autophosphorylation site domain (2E, Gullick *et al.*, 1986*b*) (Figure 1). Both antibodies however, gave consistant results. Immunoprecipitation and phosphorylation of immune complexes from tumour lysates showed concordant large variations in receptor expression (Figure 2) suggesting that the receptor was enzymatically active in these specimens. In each case preincubation of the immune complexes with a saturating concentration of EGF stimulated autophosphorylation (Figure 2) indicating that the receptor isolated from tumours 8 and 14 were activated by ligand binding. Lower exposure of the gel demonstrated that tumour 13 was also stimulated (data not shown).

It has been reported previously (Libermann *et al.*, 1985; Wong *et al.*, 1987) that the EGF receptor gene is commonly amplified in high grade gliomas. We therefore examined the EGF receptor gene structure and copy number by Southern blotting. Four of the 14 (29%) tumours examined displayed additional copies of the EGF receptor gene when compared to the single copy alpha-2-1 and alpha-1-1 collagen genes situated on chromosomes 7 and 17 respectively (Figure 3). There was no evidence of selective reduplication of chromosome 7 in any of these tumours, as determined by densitometric scanning of the autoradiographic signal produced by the alpha-2-1 and alpha-1-1 collagen gene probes. With the one restriction enzyme employed there was no sign of gene rearrangement. In several cases there was a relationship between high levels of protein expression observed by Western blotting and/or kinase activity with amplification of the EGF receptor gene (Table II). Tumour 8 and 13 however, appear to express high levels of receptor protein without evident gene amplification and tumour 9 gene amplification without grossly elevated levels of receptor protein. This discrepancy may be due to the necessity to use different fragments of the tumour in each assay system and the difficulty in reliably confirming the presence of tumour cells in each region of the biopsy.

Some reports have suggested that the EGF receptor is rarely overexpressed or its gene amplified in cell lines derived from brain tumours (Humphrey *et al.*, 1988). We therefore examined the expression of the EGF receptor in a series of 11 independently derived cell lines. Each line gave a surprisingly similar signal (data not shown) suggesting a low level of expression relative to A431 cell lysate used as a positive

**Table I** Pathological diagnosis of brain tumours and cell lines

| *Tumour code* | *Diagnosis* | *Cell line* | *Diagnosis* |
|---|---|---|---|
| 1 | Glioblastoma multiforme (GMB) | 17/81 | Astrocytoma Grade I |
| 2 | GBM | 1N/259 | Astrocytoma Grade IV |
| 3 | Malignant glioma | 1N/293 | GBM |
| 4 | Mixed Oligo-astrocytoma Grade III | 1N/353 | Malignant Glioma Grade IV |
| 5 | GBM | 1N/392 | Medulloblastoma |
| 6 | GBM | 1N/859 | GBM |
| 7 | GBM | 1N/938 | Oligodendroglioma |
| 8 | GBM | 1N/981 | Astrocytoma Grade II |
| 9 | GBM | 1N/1025 | GBM |
| 10 | Malignant astrocytoma Grade III | 1N/1056 | Astrocytoma Grade IV |
| 11 | GBM | 1N/1113 | Astrocytoma Grade I |
| 12 | GBM | | |
| 13 | GBM | | |
| 14 | Malignant glioma | | |

**Figure 1** Western blot of EGF receptors from extracts of primary brain tumours. Track 1, molecular weight markers; tracks 2–15, tumours 1–14; tracks 16–17, 1 and 3 μg A431 cell membranes.

**Figure 2** Immunoprecipitation and phosphorylation of EGF receptors from primary brain tumours. Tracks 1–5, tumours 13, 8, 12, 14, A431 membranes; tracks 6–10, tumours 13, 8, 12, 14, A431 membranes incubated with EGF. All immunoprecipitated with antibody EGFR1 to the EGF receptor.

control. Subsequently, as shown below, additional aliquots of the same cell lysates showed variable amounts of other growth factor receptors indicating that this was not an artifactual result.

### *c-*erb*B-2*

In initial experiments no c-*erb*B-2 protein expression was observed either by Western blotting or by immunoprecipitation followed by phosphorylation of the same primary tumour cell lysates. We therefore attempted to optimise the conditions of the phosphorylation reaction to increase the sensitivity of the assay. Since no ligand for the c-*erb*B-2 protein was available to stimulate phosphorylation, we examined the metal ion, time and temperature dependence of the reaction employing a chimeric protein consisting of the extracellular domain of the EGF receptor and the intracellular kinase domain to rat *neu* protein expressed at high levels in NIH3T3 cells (Lehvaslaiho *et al.*, 1989). Autophosphorylation of the construct in response to EGF was employed as an indication of receptor activation. It was found that 3 mM $MnCl_2$ gave maximal autophosphorylation but that $MgCl_2$ did not support the reaction. Combinations of the two ions did not enhance autophosphorylation further. It was also found that maximal autophosphorylation was observed at room temperature and 15 min of incubation (data not shown). Despite several subsequent attempts employing these conditions no c-*erb*B-2 protein expression was detected in the tumour extracts. When the Southern blot shown in Figure 3 was reprobed to detect the c-*erb*B-2 gene no differences in fragment pattern or intensities were observed relative to normal DNA (data not shown), indicating no detectable gene rearrangement or amplification.

It was still possible, however, that the c-*erb*B-2 protein might be expressed at low levels in the tumours. Activating mutations in the transmembrane region of the rat *neu* gene have been observed which lead to cell transformation despite low levels of protein expression (Bargmann & Weinberg, 1988). Similar mutations artifically introduced into human c-*erb*B-2 have the same effect (Segatto *et al.*, 1988). We therefore examined the sequence of the c-*erb*B-2 transmembrane region in DNA extracted from these tumours for the presence of such mutations. DNA was amplified by PCR and probed with wild type and mismatched oligonucleotides as described by Lemoine *et al.* (1990*a*). No mutations were found in this region of the gene (data not shown).

We next examined the tumour cell lines for c-*erb*B-2 expression by Western blotting. The c-*erb*B-2 protein was detected at variable but generally low levels in all of the cell lines tested. In one case, cell line 1N/392 (Figure 4, track 8), a somewhat higher level of c-*erb*B-2 protein was observed. Much less protein was seen however than present in extracts of the overexpressing breast cancer cell line SKBR-3 (Figure 4).

*PDGF receptor*

Polyclonal antipeptide antibodies were raised in rabbits to the c-terminal 13 amino acids of the mouse B-type PDGF receptor. In ELISA reactions these were shown to react with the immunising peptide and to cross react strongly and to an equal extent with the cognate sequence from the human B-type receptor and the less related sequence in the human A-type receptor (data not shown). Immunoprecipitation of $^{35}S$-methionine labelled cell lysate from the mouse cell line NR6 (known to express high levels of PDGF receptors, Bowen-Pope *et al.*, 1985) revealed the presence of two specific bands, one minor, sharp band running a 160 Kd and a major diffuse band at 185 Kd (Figure 5*a*). These are identical in size and appearance to the partially glycosylated and fully glycosylated PDGF receptor respectively (see for instance Hart *et al.*, 1987). In order to confirm the identity of these species cell lysates were immunoprecipitated and treated with or without PDGF and then autophorphosylated with $^{32}P$-gamma-ATP. Both bands were again seen and their level of phosphorylation was increased following preincubation with PDGF (Figure 5*b*). In all cases preincubation of the antibodies with the immunising peptide prevented immunoprecipitation.

**Figure 3** Southern blot of DNA from brain tumours probed for the EGF receptor. **a**, examples of EGF receptor gene amplification: track N: placental DNA; tracks 1–10, brain tumours; tracks 2 (tumour 10) and 9 (tumour 14) show amplification of 12 and 9 fold respectively of the EGF receptor gene. Track 1, tumour 11; track 3, tumour 7; track 4, tumour 6; track 5, tumour 8; track 6, tumour 12; track 7, tumour 13; track 8, tumour 12; track 10, tumour 1. **b**, Reprobed with alpha-2-1 collagen gene on chromosome 7 to normalise for loading of DNA.

**Figure 4** Western blot for c-*erb*B-2 in various brain tumour derived cells lines. Track 1, molecular weight markers; tracks 2–16, cell lines 17/18, 1N/259, 1N/293, 1N/293, U-251, 1N/353, 1N/392, U-251, U-251, 1N/859, 1N/938, 1N/981, 1N/1025, 1N/1056, 1N/1113; tracks 17 and 18, 5 and 10 µg of SKBR-3 cell lysate.

**Figure 5** **a**, Immunoprecipitation of PDGF receptor from NR6 mouse fibroblasts labelled with $^{35}S$ methionine. Track 1, 5 µl antibodies to the PDGF receptor; track 2, 5 µl antibodies to the PDGF receptor preincubated with immunizing peptide. **b**, Immunoprecipitation and phosphorylation of PDGF receptors from NR6 cells. Tracks 1 and 3, antibody to PDGF receptor; tracks 2 and 4, preincubated with peptide; tracks 3 and 4 plus PDGF; tracks 1 and 2, minus PDGF.

**Table II** Summary of receptor protein expression, kinase activity and gene copy number

| | *EGF-Receptor* | | | *PDGF-R* | | | | |
|---|---|---|---|---|---|---|---|---|
| *Tumour* | *Western blot* | *Southern blot* | *Immpt/ Phos.* | *Immpt/ Phos.* | *Cell line* | *EGFR Western* | *c-erbB-2 Western* | *PDGFR Western* |
| 1 | + | − | N/A | + | 17/81 | + | + + | + |
| 2 | + + | − | N/A | + + | 1N/259 | + | + | ± |
| 3 | − | − | N/A | − | 1N/293 | + | + + | + |
| 4 | − | − | N/A | + | 1N/353 | + | + + | + |
| 5 | + + + | 13 × | N/A | − | 1N/392 | + | + + + | − |
| 6 | + | − | N/A | − | 1N/859 | + | + + | ± |
| 7 | + | − | N/A | + | 1N/938 | + | + + | + |
| 8 | + + | − | + + + | + + | 1N/981 | + | + + | + |
| 9 | − | 5 × | N/A | − | 1N/1025 | + | + + | + |
| 10 | + + | 12 × | N/A/ | + + | 1N/1056 | + | + | − |
| 11 | + | − | N/A/ | + + + | 1N/1113 | + | + + | + + |
| 12 | + | − | − | + + | | | | |
| 13 | + + | − | + + + | + + + | | | | |
| 14 | + + | 9 × | + | + | | | | |

Brain tumour lysates were immunoprecipitated with antibodies to the PDGF receptor and the immune complexes phosphorylated. Autoradiography revealed that several tumours contained detectable levels of receptors (Figure 6, Table II) which were similar in size to the receptor recognised in the human cell line AG1523 (Figure 6, tracks 16–18). Several other strongly labelled bands were also observed. However, these were present even following preincubation of the antibody with the immunising peptide. It seems likely that these are present non-specifically in the immunoprecipitates and became labelled upon the addition of ATP. We have not investigated their identity further. Western blotting of the same tumours did not, however, detect PDGF receptor expression suggesting that the phosphorylation technique is much more sensitive. Western blotting of tumour cell lines did show expression of PDGF receptors at variable but barely detectable levels (data not shown) supporting this premise.

### Discussion

The EGF receptor is expressed at low levels in the normal human brain. Some conflict exists however as to its relative expression on neurons, glial cells and astrocytes. Immunohistochemical staining demonstrated receptors on many types of nerve cells but not on astrocytes and glial cells (Werner *et al.*, 1988). Direct binding of iodinated EGF to primary cultures of rat brain cells, however, demonstrated higher levels of receptors on glial cells than on neurons (Wang *et al.*, 1989). Several reports have indicated that vastly increased numbers of receptors are frequently found on certain human brain tumour cells. In this work we confirm that the EGF receptor gene is commonly amplified in glioblastomas of high grade leading to greatly elevated levels of receptor expression. The incidence of 29% of amplification in this small study is consistent with that reported previously (Libermann *et al.*, 1985; Wong *et al.*, 1987). With the one restriction enzyme employed we saw no evidence of gene rearrangement in these specimens. Other have reported rearrangements to occur quite frequently in amplified cases (Humphrey *et al.*, 1988 (5/6); Libermann *et al.*, 1985 (2/4); Wong *et al.*, 1987 (6/14); Yamazaki *et al.*, 1988 (2/2)). Further studies with a panel of restriction endonucleases would be required to more fully exclude the possiblity of rearrangements in our cases. Amplification led to high levels of EGF receptor protein expression which was capable of autophosphorylation indicating that it was enzymatically active. Yamazaki *et al.* (1988) have found that in two cases of glioblastoma multiforme where there was an amplified, rearranged EGF receptor gene, the protein product was consitutively active in that addition of EGF to an immune complex kinase assay did not promote additional autophosphorylation. In our immunoprecipitation kinase assays however, in each case addition of EGF did promote receptor autophosphorylation demonstrating that the receptors were not fully activated. Conversely, a report has appeared in which the EGF receptor gene was found to be amplified in the human glioblastoma cell line SF268, but the protein expressed was enzymatically inactive (Wells *et al.*, 1988). In another publication this line bound EGF which could be crosslinked to a protein of the same size as the EGF receptor, but the cells were unresponsive to EGF (Westphal *et al.*, 1985). In this study we found uniformly low levels of expression of EGF receptor protein in all the cell lines studied. It is possible that overexpression of EGF receptors in brain tumours is a selective advantage *in vivo*, but a disadvantage to cells in culture (Humphrey *et al.*, 1988) and that mutation may have occurred in the EGF receptor gene in the SF268 cell line which inactivates the receptors catalytic activity (Wells *et al.*, 1988).

Overexpression of EGF receptors in breast cancer is associated with poor prognosis (Sainsbury *et al.*, 1987). It is not known whether overexpression in brain tumours defines a subgroup of tumours with different biological characteristics. Study of this is currently hampered by the lack of immunological reagents which reliably detect EGF receptor expression in paraffin embedded archival material. The high levels of receptor expression do however provide a target for immunoscintigraphy (Takahashi *et al.*, 1987) and immunotherapy (Epenetos *et al.*, 1985; Kalofonos *et al.*, 1989) and novel forms of receptor inhibitors (Gullick, 1990).

We found no evidence of aberrant expression or gene amplification of c-*erb*B-2 in any of the primary tumour biopsies. Amplification and overexpression of this gene occurs in about 20% of breast (Slamon *et al.*, 1989; Gullick *et al.*, 1990), stomach (Falck & Gullick, 1989) and ovarian cancers (Slamon *et al.*, 1989) and with a lower frequency in other tumour types (Hall *et al.*, 1990; Yokota *et al.*, 1986; Lemoine *et al.*, 1990*b*). The c-*erb*B-2 protein, detected by Western blotting, was expressed at low, variable levels in the cell lines, but not in the primary tumour biopsies. It may be that it is also expressed in the primary tumours, but at levels below our limit of detection. c-*erb*B-2 protein is normally expressed in some areas of foetal (Quirke *et al.*, 1989) and adult (Quirke & Gullick, unpublished results) human brain.

We can conclude that amplification and overexpression of the c-*erb*B-2 protein is probably a rare event in human brain tumours, if it occurs at all. However, it was possible that low level expression of a mutant c-*erb*B-2 protein could occur and we therefore examined the gene for mutations at position 659 in the amino acid sequence. Previously this has been shown to occur in a rat carcinogenesis model leading to tumours of the CNS (Bargmann & Weinberg, 1988) and an equivalent change can convert the human gene to a powerful oncogene (Segatto *et al.*, 1988). Using PCR and mismatched oligonucleotide hybridisation we saw no evidence of mutations in this region of the gene. Previously we have also found no mutations in a large series of breast (Lemoine *et al.*, 1990*a*), pancreatic (Hall *et al.*, 1990) and thyroid tumours (Lemoine *et al.*, 1990*b*). It is apparent that this change may be confined to the rat chemical carcinogenesis model.

The tumours were also examined for the expression of the PDGF receptor protein. The detailed distribution of PDGF receptors in the human brain is not known. Receptors are, however, expressed on normal cultured human glial cells (Heldin *et al.*, 1981), astrocytes (Richardson *et al.*, 1988) and rat neurons (Smits *et al.*, 1990). Receptors are also present on transformed cell lines derived from gliomas (Nister *et al.*, 1986; Nister *et al.*, 1988). In addition, several studies have demonstrated the production of PDGF A chain and PDGF B chain by cultured glial cells (Betsholtz *et al.*, 1986) and neuroblastoma cells (van Zoelen *et al.*, 1985) and in primary brain tumours (Hermanssen *et al.*, 1988). A characteristic of

**Figure 6** Immunoprecipitation and phosphorylation of PDGF receptors from primary brain tumours. Tracks 1–3, tumour 7; tracks 4–6, tumour 8; tracks 7–9, tumour 9; tracks 10–12, tumour 10; tracks 13–15, human foreskin fibroblasts; tracks 16–18, AG1523 cells. Those tracks indicated + PEP represent immunoprecipitates in which immunising peptide has been added as a competitive inhibitor of immunoprecipitation.

high grade gliomas is the large amount of reactive endothelial cells present. Human umbilical vein endothelial cells do not appear to possess PDGF receptors, but they do secrete PDGF (Dicorleto & Bowen-Pope, 1983; Barrett *et al.*, 1984; Collins *et al.*, 1985) although this may be slightly different in structure to that made by glial cells (Tong *et al.*, 1987). Interestingly however, *in situ* hybridisation of three cases of glioblastoma multiforme revealed quite high levels of apparent PDGF receptor mRNA in proliferating tumour associated endothelial cells suggesting a possible autocrine or paracrine growth mechanism (Hermansson *et al.*, 1988). In this study we show that the PDGF receptor protein was present in several primary tumour extracts and was catalytically active. It was also present at low, variable levels in many of the cell lines examined indicating that the receptor was present in tumour cells. No reports have ever indicated that the PDGF receptor gene is amplified in any human tumours.

The critical question is whether either the production of PDGF or its receptors is abnormal in the tumours and is promoting their transformed phenotype. Detailed studies of the relative levels of receptor and ligand expression by normal and transformed cells are required to address this point. It seems however that there is circumstantial evidence for their involvement.

In summary, the EGF receptor gene is amplified and overexpressed in human brain tumours. From this work, however, it appears that although the c-*erb*B-2 protein is expressed in brain tumour cell lines, there is no evidence that this expression is abnormal. PDGF receptor expression could be demonstrated in human brain tumours and it may be that the PDGF system is influential in tumour cell growth regulation, but more experiments are required to address this issue.

We are grateful to Miss Zoë Redley for preparing this manuscript.

### References

BARGMANN, C.I. & WEINBERG, R.A. (1988). Oncogenic activation of the *neu*-encoded receptor protein by point mutation and deletion. *EMBO J.*, **7**, 2043.

BARRETT, T.B., GAJDUSEK, C.M., SCHWARTZ, S.M., MCDOUGALL, J.K. & BENDITT, E.P. (1984). Expression of the *sis* gene by endothelial cells in culture and *in vivo*. *Proc. Natl Acad. Sci. USA*, **81**, 6772.

BETSHOLTZ, C., JOHNSSON, A., HELDIN, C.-H. & 9 others (1986). cDNA sequence and chromosomal localization of human platelet-derived growth factor A-chain and its expression in tumour cell lines. *Nature*, **320**, 695.

BOWEN-POPE, D.F., ROSENFELD, M.E., SEIFERT, R.A. & ROSS, R. (1985). The platelet-derived growth factor receptor. *Int. J. Neuroscience*, **26**, 141.

BRADFORD, M.M. (1976). A rapid and sensitive method for the quantitation of microgram quantities of protein utilizing the principle of protein dye binding. *Anal. Biochem.*, **72**, 248.

CLAESSON-WELSH, L., RONNSTRAND, L. & HELDIN, C.-H. (1987). Biosynthesis and intracellular transport of the receptor for platelet-derived growth factor. *Proc. Natl Acad. Sci. USA*, **84**, 8796.

COLLINS, T., GINSBURG, D., BOSS, J.M., ORKIN, S.H. & POBER, J.S. (1985). Cultured human endothelial cells express platelet-derived growth factor B chain: cDNA cloning and structural analysis. *Nature*, **316**, 748.

CORBETT, I.P., HENRY, J.A., ANGUS, B. & 8 others (1990). NCL-CB11 a new monoclonal antibody recognising the internal domain of the c-*erb*B-2 oncoprotein effective for use on formalin fixed paraffin embedded tissue. *J. Pathology*, **161**, 15.

DICORLETO, P.E. & BOWEN-POPE, D.F. (1983). Cultured endothelial cells produce a platelet-derived growth factor-like protein. *Proc. Natl Acad. Sci. USA*, **80**, 1919.

EPENETOS, A.A., COURTENAY-LUCK, N.S., PICKERING, D. & 4 others (1985). Antibody guided irradiation of brian glioma by arterial infusion of radioactive monoclonal antibody against epidermal growth factor receptor and blood group A antigen. *Br. Med. J.*, **290**, 1463.

FALCK, V.G. & GULLICK, W.J. (1989). c-*erb*B-2 oncogene product staining in gastric adenocarcinoma. An immunohistological study. *J. Pathol.*, **159**, 107.

FRANKS, A.J. & BURROW, H.M. (1986). *In vitro* heterogeneity in human gliomas: are all transformed cells of glial origin? *Anticancer Res.*, **6**, 625.

GRONWALD, R.G.K., GRANT, F.J., HALDEMAN, B.A. & 6 others (1988). Cloning and expression of a cDNA coding for the human platelet-derived growth factor receptor: evidence for more than one receptor class. *Proc. Natl Acad. Sci. USA*, **85**, 3435.

GULLICK, W.J. (1990). Inhibitors of growth factor receptors. In *Genes and Cancer*, Carney, D. & Sikora, K. (eds) p. 263. John Wiley: Chichester.

GULLICK, W.J., BERGER, M.S., BENNETT, P.L.P., ROTHBARD, J.B. & WATERFIELD, M.D. (1987). Expression of the c-*erb*B-2 protein in normal and transformed cells. *Int. J. Cancer*, **40**, 246.

GULLICK, W.J., DOWNWARD, J., FOULKES, J.G. & WATERFIELD, M.D. (1986*a*). Antibodies to the ATP-binding site of the human epidermal growth factor receptor as specific inhibitors of EGF-stimulated protein tyrosine kinase activity. *Eur. J. Biochem.*, **158**, 245.

GULLICK, W.J., DOWNWARD, J. & WATERFIELD, M.D. (1985). Antibodies to the auto-phosphorylation sites of the epidermal growth factor receptor protein tyrosine kinase as probes of structure and function. *EMBO J.*, **4**, 2869.

GULLICK, W.J., LOVE, S.B., WRIGHT, C. & 4 others (1990). c-*erb*B-2 protein overexpression in breast cancer is a risk factor in patients with involved and uninvolved lymph nodes. *Br. J. Cancer*, (in the press).

GULLICK, W.J., MARSDEN, J.J., WHITTLE, N., WARD, B., BOBROW, L. & WATERFIELD, M.D. (1986*b*). Expression of epidermal growth factor receptors on cervical, ovarian and vulval carcinomas. *Cancer Res.*, **46**, 285.

GULLICK, W.J., TUZI, N.L., KUMAR, S., PATERSON, H., QUIRKE, P. & GULLICK, W.J. (1989). c-*erb*B-2 and c-myc genes and their expression in normal tissues and in human breast cancer. *Cancer Cells*, **7**, 393.

GULLICK, W.J. & VENTER, D.J. (1989). The c-*erb*B-2 gene and its expression in human cancers. In *The Molecular Biology of Cancer*, Sikora, K. & Waxman, J. (eds) p. 38. Blackwell: Oxford.

HALL, P.A., HUGHES, C.M., STADDON, S.L., RICHMAN, C.I., GULLICK, W.J. & LEMOINE, N.R. (1990). The c-*erb*B-2 proto-oncogene in human pancreatic cancer. *J. Pathol.*, **161**, 195.

HARSH, G.R., ROSENBLUM, M.L. & WILLIAMS, L.T. (1989). Oncogene-related growth factors and growth factor receptors in human malignant glioma-derived cell lines. *J. Neurol. Oncol.*, **7**, 47.

HART, C.E., SEIFERT, R.A., ROSS, R. & BOWEN-POPE, D.F. (1987). Synthesis, phosphorylation and degradation of multiple forms of the platelet-derived growth factor receptor studied using a monoclonal antibody. *J. Biol. Chem.*, **262**, 10780.

HELDIN, C.-H., WESTERMARK, B. & WASTESON, A. (1981). Specific receptors for platelet-derived growth factor on cells derived from connective tissue and glia. *Proc. Natl Acad. Sci. USA*, **78**, 3664.

HERMANSSON, M., NISTER, M., BETSHOLTZ, C., HELDIN, C.-H., WESTERMARK, B. & FUNA, K. (1988). Endothelial cell hyperplasia in human glioblastoma: coexpression of mRNA for platelet-derived growth factor (PDGF) B chain and PDGF receptor suggests autocrine growth stimulation. *Proc. Natl. Acad. Sci. USA*, **85**, 7748.

HUMPHREY, P.A., WONG, A.J., VOGELSTEIN, B. & 4 others (1988). Amplification and expression of the epidermal growth factor receptor gene in human glioma xenografts. *Cancer Res.*, **48**, 2231.

KALOFONOS, H.P., PAWLIKOWSKA, T.R., HEMINGWAY, A. & 9 others (1989). Antibody guided diagnosis and therapy of brain gliomas using radiolabelled monoclonal antibodies against epidermal growth factor receptor and placental alkaline phosphatase. *J. Nucl. Med.*, **30**, 1636.

KRAUS, M.H., POPESCU, N.C., AMSBAUGH, S.C. & KING, C.R. (1987). Overexpression of the EGF receptor-related proto-oncogene *erb*B-2 in human mammary tumour cell lines by different molecular mechanisms. *EMBO J.*, **6**, 605.

LEHVASLAIHO, H., LEHTOLA, L., SISTONEN, L. & ALITALO, K. (1989). A chimeric EGF-R-*neu* proto-oncogene allows EGF to regulate *neu* tyrosine kinase and cell transformation. *EMBO J.*, **8**, 159.

LEMOINE, N.R., STADDON, S.L., DICKSON, C., BARNES, D.M. & GULLICK, W.J. (1990*a*). Absence of activating transmembrane mutations in the c-*erb*B-2 proto-oncogene in human breast cancer. *Oncogene*, **5**, 237.

LEMOINE, N.R., WYLLIE, F.S., LILLEHAUG, J.R. & 8 others (1990*b*). Absence of abnormalities of the c-*erb*B-1 and c-*erb*B-2 proto-oncogenes in human thyroid neoplasia. *Eur. J. Cancer*, **26**, 777.

LIBERMANN, T.A., NUSBAUM, H.R., RAZON, N. & 7 others (1985). Amplification, enhanced expression and possible rearrangement of EGF receptor gene in primary human brain tumours of glial origin. *Nature*, **313**, 144.

MATSUI, T., HEIDARAN, M., MIKI, T. & 5 others (1989). Isolation of a novel receptor cDNA establishes the existence of two PDGF receptor genes. *Science*, **243**, 800.

NISTER, M., HELDIN, C.-H., & WESTERMARK, B. (1986). Clonal variation in the production of a platelet-derived growth factor-like protein and expression of corresponding receptors in a human malignant glioma. *Cancer Res.*, **46**, 332.

NISTER, M., LIBERMANN, T.A., BETSHOLTZ, C. & 5 others (1988). Expression of messenger RNAs for platelet-derived growth factor and transforming growth factor-alpha and their receptors in human malignant glioma cell lines. *Cancer Res.*, **48**, 3910.

OZANNE, B., RICHARDS, C.S., HENDLER, F., BURNS, D. & GUSTERSON, B. (1986). Overexpression of the EGF receptor is a hallmark of squamous cell carcinomas. *J. Pathol.*, **149**, 9.

QUIRKE, P., PICKLES, A., TUZI, N.L., MOHAMDEE, O. & GULLICK, W.J. (1989). Pattern of expression of c-*erb*B-2 oncoprotein in human fetuses. *Br. J. Cancer*, **60**, 64.

RICHARDSON, W.D., PRINGLE, N., MOSLEY, M.J., WESTERMARK, B. & DUBOIS-DALCQ, M. (1988). A role for platelet-derived growth factor in normal gliogenesis in the central nervous system. *Cell*, **53**, 309.

RUSSEL, D.S. & RUBINSTEIN, L.J. (1977). In *Pathology of Tumours of the Nervous System*, 4th edition, Edward Arnold: London.

SAINSBURY, J.R.C., FARNDON, J.R., NEEDHAM, G.K., MALCOLM, A.J. & HARRIS, A.L. (1987). Epidermal growth factor receptor status as predictor of early recurrence of and death from breast cancer. *Lancet*, **i**, 1398.

SEGATTO, O., KING, C.R., PIERCE, J.H., DI FIORE, P.P. & AARONSON, S.A. (1988). Different structural alterations upregulate in vitro tyrosine kinase activity and transforming potency of the *erb*B-2 gene. *Mol. Cell. Biol.*, **8**, 5570.

SLAMON, D.J., GODOLPHIN, W., JONES, L.A. & 8 others (1989). Studies of the HER-2/*neu* proto-oncogene in human breast and ovarian cancer. *Science*, **244**, 707.

SMITS, A., WESTERMARK, B., HELDIN, C.-H. & FUNA, K. (1990). Presence of PDGF beta-receptors on primary cultures of rat brain neurons. *EMBL Conference 'Oncogenes and Growth Control'*. Abstract 62.

TAKAHASHI, H., HERLYN, D., ATKINSON, B. & 5 others (1987). Radioimmunodetection of human glioma xenografts by monoclonal antibody to epidermal growth factor receptor. *Cancer Res.*, **47**, 3847.

TONG, B.D., AUER, D.E., JAYE, M. & 5 others (1987). cDNA clones reveal differences between human glial and endothelial cell platelet-derived growth factor A-chains. *Nature*, **328**, 619.

VAN ZOELEN, E.J.J., VAN DE VEN, W.J.M., FRANSSEN, H.J. & 4 others (1985). Neuroblastoma cells express c-*sis* and produce a transforming growth factor antigenically related to the platelet-derived growth factor. *Mol. Cell. Biol.*, **5**, 2289.

VENTER, D.J., TUZI, N.L., KUMAR, S. & GULLICK, W.J. (1987). Overexpression of the c-*erb*B-2 oncoprotein in human breast carcinomas: immunohistochemical assessment correlates with gene amplification. *Lancet*, **ii**, 69.

WANG, S.-L., SHIVERICK, K.T., OGILVIE, S., DUNN, W.A. & RAIZADA, M.K. (1989). Characterization of epidermal growth factor receptors in astrocytic glial and neuronal cells in primary culture. *Endocrinol.*, **124**, 240.

WATERFIELD, M.D., MAYES, E.L.V., STROOBANT, P. & 5 others (1982). A monoclonal antibody to the human epidermal growth factor receptor. *J. Cell. Biochem.*, **20**, 149.

WELLS, A., BISHOP, J.M. & HELMESTE, D. (1988). Amplified gene for the epidermal growth factor receptor in a human glioblastoma cell line encodes an enzymatically inactive protein. *Mol. Cell. Biol.*, **8**, 4561.

WERNER, M.H., NANNEY, L.B., STOSCHECK, C.M. & KING, L.E. (1988). Localization of immunoreactive epidermal growth factor receptors in human nervous system. *J. Histochem. Cytochem.*, **36**, 81.

WESTPHAL, M., HARSH, G.R., ROSENBLUM, M.L. & HAMMONDS, R.G. (1985). Epidermal growth factor receptors in the human glioblastoma cell line SF268 differ from those in epidermoid carcinoma cell line A431. *Bioc. Biop. Res. Comm.*, **132**, 284.

WONG, A.J., BIGNER, S.H., BIGNER, D.D., KINZLER, HAMILTON, S.R. & VOGELSTEIN, B. (1987). Increased expression of the epidermal growth factor receptor gene in malignant gliomas is invariably associated with gene amplification. *Proc. Natl Acad. Sci. USA*, **84**, 6899.

WRIGHT, J.A., SMITH, H.S., WATT, F.M., HANCOCK, M.C., HUDSON, D.L. & STARK, G.R. (1990). DNA amplification is rare in normal human cells. *Proc. Natl Acad. Sci. USA*, **87**, 1791.

YAMAZAKI, H., FUKUI, Y., UEYAMA, Y. & 4 others (1988). Amplification of the structurally and functionally altered epidermal growth factor receptor gene (c-*erb*B) in human brain tumours. *Mol. Cell. Biol.*, **8**, 1816.

YARDEN, Y., ESCOBEDO, J.A., KUANG, W.-J. & 10 others (1986). Structure of the receptor for platelet-derived growth factor helps define a family of closely related growth factor receptors. *Nature*, **323**, 226.

YOKOTA, J., YAMAMOTO, T., TOYOSHIMA, K. & 4 others (1986). Amplification of c-*erb*B-2 ocogene in human adenocarcinomas *in vivo*. *Lancet*, **i**, 765.